# Microglial Immunoreceptor Tyrosine-Based Activation and Inhibition Motif Signaling in Neuroinflammation

**DOI:** 10.4061/2010/587463

**Published:** 2010-06-22

**Authors:** Bettina Linnartz, Yiner Wang, Harald Neumann

**Affiliations:** Neural Regeneration, Institute of Reconstructive Neurobiology, University Hospital Bonn, University Bonn, 53127 Bonn, Germany

## Abstract

Elimination of extracellular aggregates and apoptotic neural membranes without inflammation is crucial for brain tissue homeostasis. In the mammalian central nervous system, essential molecules in this process are the Fc receptors and the DAP12-associated receptors which both trigger the microglial immunoreceptor tyrosine-based activation motif- (ITAM-) Syk-signaling cascade. Microglial triggering receptor expressed on myeloid cells-2 (TREM2), signal regulatory protein-*β*1, and complement receptor-3 (CD11b/CD18) signal via the adaptor protein DAP12 and activate phagocytic activity of microglia. Microglial ITAM-signaling receptors are counter-regulated by immunoreceptor tyrosine-based inhibition motif- (ITIM-) signaling molecules such as sialic acid-binding immunoglobulin superfamily lectins (Siglecs). Siglecs can suppress the proinflammatory and phagocytic activity of microglia via ITIM signaling. Moreover, microglial neurotoxicity is alleviated via interaction of Siglec-11 with sialic acids on the neuronal glycocalyx. Thus, ITAM- and ITIM-signaling receptors modulate microglial phagocytosis and cytokine expression during neuroinflammatory processes. Their dysfunction could lead to impaired phagocytic clearance and neurodegeneration triggered by chronic inflammation.

## 1. Microglia and Alzheimer's Disease

Microglial cells originate from myeloid cells of the hematopoietic lineage and are the resident immune cells of the central nervous system (CNS). They are involved in the active immune defense by their ability to phagocytose invading bacteria and to release reactive oxygen species acting as microbicides. In the healthy brain, microglia are relative evenly distributed and predominantly found in a so-called “resting” state, displaying a small cell body with many highly branched processes, which are highly motile and continuously monitor the brain parenchyma [1–3]. Microglia are involved in tissue maintenance, execution of innate immunity, and participation in adaptive immune responses [1–3]. They are regarded as active sensors, searching for and reading biochemical signals of pathogenic changes in the brain environment [2]. In response to injury, ischemia and inflammatory stimuli microglia change from an immunologically silent state to an activated state that is reflected in different morphological appearance - amoeboid, rodlike, or phagocytic. They can migrate to the site of disturbance, secrete a wide range of soluble factors including cytokines as well as neurotrophic factors, and phagocytose cellular debris. Thereby, microglia contribute to tissue homeostasis and regeneration [2–5]. The effects of activated microglia can be highly diverse. On the one hand, they are neurotoxic by producing pro-inflammatory mediators including cytokines and reactive oxygen species such as interleukin-1*β*, tumor necrosis factor-*α* and nitric oxide, which are potent inducers of neuronal damage and cell death [3]. On the other hand, they can also initiate anti-inflammatory and immunosuppressive signaling that results in repair, resolution of inflammation and turning back to tissue homeostasis [3, 6, 7]. Furthermore, microglia act as regulators of neuronal survival and development through cytokines and chemokines such as interleukin-6 and CCL5 (RANTES). Activated, interleukin-6 producing microglia have been shown to decrease *in vitro *the neurogenesis of neural stem cells and increase the number of apoptotic cells in differentiating cultures [8]. Moreover, upon stimulation, RANTES is produced by microglia [9]. Due to the observation that similar amounts of RANTES are produced by fetal and adult microglia, Hu et al. suggest this chemokine to be acquired early in brain development [9].

Increasing evidence indicates that microglia are involved in almost all types of brain pathology. In the aging brain and most chronic neurodegenerative diseases including Parkinson's disease and Alzheimer's disease (AD), microglial cells become activated and provoke ambivalent effects. They can either be deleterious by enhancing neurodegeneration through secreting cytokines and neurotoxins [10], or might be beneficial by principally migrating to the amyloid-*β* (A*β*) plaques and phagocytosing A*β* deposits. Recently, it was shown by *in vivo* multiphoton microscopy in different animal models of AD that A*β* plaques could appear within 24 hours and microglial cells are activated and recruited to the newly formed plaques within one day [11]. Additionally, within one week after the onset of plaque formation dysmorphic neurites were present [11]. Interestingly, microglial cells seem to contribute to AD progression. Although maintaining their ability to produce pro-inflammatory cytokines, microglia of aging APP/PS1-transgenic mice, a mouse model of A*β* plaque formation associated with AD, become dysfunctional and display a reduced A*β* clearance capability [12], suggesting that A*β* plaques might partially result from impaired microglial removal. However, in APP-transgenic mice that barely exhibit resident microglia, formation and maintenance of A*β* plaques have been lately demonstrated unchanged [13]. Nevertheless, several lines of *in vitro* evidence suggest the involvement of innate immune signaling during recognition of A*β*. Different receptors expressed on microglia such as CD14 and toll-like receptors (TLR) 2 and 4 are known to contribute to the clearance of A*β* plaques in AD [14–16]. CD14 is involved in the uptake of the bacterial component lipopolysaccharide (LPS) [15]. To transduce activation signals, CD14 interacts with TLR2 and TLR4 containing dimeric complexes [16, 17]. Additionally, CD14 has been shown to specifically mediate A*β* phagocytosis *in vitro*. Cells expressing CD14 internalized significantly higher amounts of A*β* compared to CD14-deficient cells while the uptake of microbeads was unaffected [15]. Moreover, CD14 acts together with TLR2 and TLR4 to bind A*β* and subsequently activate intracellular signaling leading to phagocytosis *in vitro*. Cells deficient for either CD14, TLR2 or TLR4 could not initiate the cascades inducing phagocytosis [16]. Recently, CD36, another coreceptor of TLRs, has been described *in vitro* to facilitate the assembly of a heteromeric complex of CD36, TLR4, and TLR6 upon binding of A*β* [14]. However, the exact receptors which might scavenge A*β* and/or induce microglial phagocytic responses and signaling pathways that impair microglial phagocytosis *in vivo* are still unclear.

## 2. Microglial ITAM-ITIM Signaling

**Figure 1:**
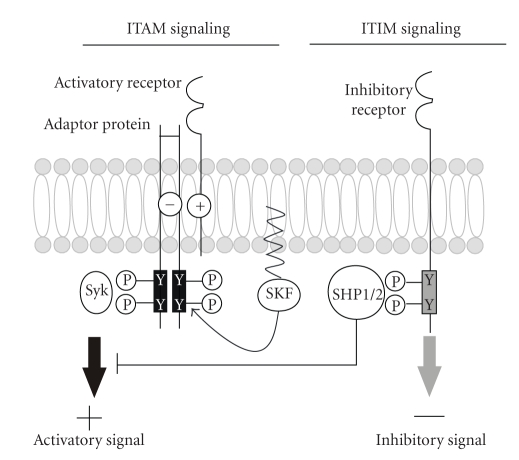
ITAM-/ITIM-signaling cascade. *Left side: *Upon ligand binding, activatory receptors like TREM2, SIRP*β*1, Fc*γ*RI or Fc*γ*RIIIA associate with ITAM containing adaptor proteins such as DAP12 or the common *γ* chains through interactions between charged amino acids (−/+) within the transmembrane regions of each protein. Subsequently, members of Src kinase family (SKF) phosphorylate tyrosine residues of ITAMs. Phosphotyrosine residues are docking sites for Syk protein kinases that upon activation mediate cellular activation via a number of downstream cascades. *Right side: *Upon ligand binding, inhibitory receptors like most Siglecs recruit SHP1 and SHP2 which can in turn terminate intracellular signals emanating from ITAM receptors.

Latest publications indicate that immunoreceptor tyrosine-based activation motif (ITAM) signaling plays an important role in the phagocytic process. ITAM-containing signaling adaptor proteins are associated with receptor subunits. After the binding of ligand and receptor, the tyrosine residues of the ITAMs become phosphorylated by members of the Src kinase family (Figure 1, left side). These phosphotyrosine residues are docking sites for Src homology 2 (SH2) domains of Syk protein kinases which upon activation mediate cellular activation via a number of downstream cascades [18–20]. The processes involved in phagocytosis of apoptotic material are well conserved from worms to mammals [21]. Draper is a phagocytic receptor of the fruit fly *Drosophila *with an ITAM in the intracellular domain. Recently, it has been described that upon phosphorylation of ITAM tyrosine residues, Draper can bind the nonreceptor tyrosine kinase Shark which is similar to the mammalian Syk. Moreover, not only the activity of Shark but also the ITAM-phosphorylation of Draper is required for Draper-mediated signaling events such as the attraction of glial membranes to damaged axons and the glial phagocytic activity [21]. Interestingly, the Draper-ITAM signaling pathway of *Drosophila* and the DAP12-ITAM signaling of mammalian immunoreceptors have a lot in common. The mammalian DAP12 molecule is a transmembrane adaptor protein that contains two ITAMs. It is expressed by microglia and associates with cell membrane receptors such as triggering receptor expressed on myeloid cells 2 (TREM2) [18] or signal regulatory protein-*β*1 (SIRP*β*1) [22]. Stimulation of SIRP*β*1 or TREM2 occurs by yet unknown endogenous ligands. For TREM2 it has been suggested that it binds to lipo-oligosaccharides of Gram-positive and -negative bacteria. The observation that the binding can be disrupted by anionic carbohydrates led to the suggestion that a charge-dependent ligand recognition takes place [23]. Upon stimulation of SIRP*β*1 or TREM2, a phosphorylation of DAP12-ITAM is induced and the phagocytic activity of microglial cells is increased *in vitro *[22, 24, 25]. TREM2-DAP12 signaling via ITAM also promotes phagocytosis of bacteria. It has been demonstrated *in vitro *that TREM2 mediates binding of bacteria and promotes their internalization dependent on Src kinase mediated tyrosine phosphorylation [26]. ITAMs are counter-regulated by immunoreceptor tyrosine-based inhibition motifs (ITIMs; Figure 1, right side). Upon ligand binding, inhibitory receptors with ITIMs prevent the activation signals that originate from receptors associated with ITAMs through the recruitment of SH2 domain containing tyrosine phosphatases (SHP1 and SHP2) which in turn can modulate the function of various signaling pathways [27, 28]. Most CD33-related sialic acid-binding immunoglobulin superfamily lectins (Siglecs), a subgroup of the immunoglobulin superfamily that recognizes sialic acid residues of glycoproteins and glycolipids, have one or more ITIMs in the cytoplasmic domain [27, 29]. Binding of Siglecs to highly sialylated proteins and lipids such as clusterin, apolipoprotein E and gangliosides that are abundantly present in AD plaques could in turn mediate an inhibitory signaling cascade. Thereby, microglial phagocytosis is possibly suppressed and the AD plaques might be left untouched [28].

## 3. Microglial Fc Receptors

An important group of receptors on the surface of phagocytes, which signal via ITAM-Syk signaling and mediate phagocytosis function, include the Fc receptors (FcR). FcR interact with the Fc part of immunoglobulin (Ig) G bound to antigen presented on microbial pathogens or autoantigens [20, 30, 31]. Except for the human Fc*γ*RIIA (CD32a), which itself possesses an ITAM located in the cytoplasmic region, activating FcRs like Fc*γ*RI (CD64) and Fc*γ*RIIIA (CD16a) have to interact with adaptor molecules, the common *γ* chain of FcR that contain the required ITAMs. The common *γ* chain of FcR is a homolog of the adaptor protein DAP12 and functionally close related to it. Subsequently, tyrosine residues of the ITAM are phosphorylated by members of the Src kinase family resulting in the establishment of docking sites for Syk kinases (Figure 1, left side). Activated Syk kinases in turn initiate a variety of downstream signals mediated through calcium, protein kinase C, phospholipase A2, phosphatidyl-inositol 3-kinase, extracellular signal-regulated kinase and GTPases of the Rho family leading to phagocytosis of IgG coated and opsonized particles and antigens [31–33]. Accordingly, microglial cells express the activating FcRs CD16, CD32 and CD64 and phagocytose antigens via the corresponding IgG subtypes [34, 35]. Moreover, in brain areas displaying neurodegeneration such as multiple sclerosis lesions, the expression of those FcRs on microglia is increased [34], suggesting a role of FcRs in protecting the surrounding tissue from IgG-opsonized antigens [35]. Furthermore, there is an ongoing discussion whether FcRs play a role in AD by contributing to microglial A*β* clearance [36]. In APP-transgenic mice it has been demonstrated that antibodies directed against A*β* can enter the CNS [36]. One study described that immunization of APP-transgenic mice with A*β*1-42, which induced A*β*1-42 specific antibodies, reduced A*β* deposition regardless of whether the mice were genetically deficient of the FcR domain FcR*γ*. The authors suggest that FcR-mediated mechanisms are irrelevant for the effectiveness of A*β* immunotherapy *in vivo* [37]. However, another study clearly demonstrated by using an *ex vivo *assay, in which primary microglial cells were cultured with unfixed cryostat sections of AD brains, that A*β* antibodies could evoke FcR-mediated microglial phagocytosis of A*β* plaques and subsequent A*β* degradation [36].

## 4. Microglial DAP12 Associated Receptors

Several DAP12 associated receptors are known including activating natural killer cell receptors, like KIR2DS and NKG2D, and myeloid receptors, such as signal regulatory protein-*β*1 (SIRP*β*1), TREM1, -2, -3 [38], complement receptors [39], and certain Siglecs, such as Siglec-16 [40]. This review will focus on some of them.

### 4.1. TREM2

 The glycoprotein TREM2 is expressed on microglia [41], and consists of one extracellular Ig-like domain, a transmembrane region with a charged lysine residue and a short cytoplasmic tail [18]. As TREM2 lacks an intracellular signaling tail, it is completely dependent on the presence of the adaptor protein DAP12 [18, 42]. As mentioned before, the mammalian adaptor molecule DAP12 is another protein besides the common *γ* chain of FcR that activates an ITAM-Src kinase signaling pathway. Via signaling through the adaptor protein DAP12, TREM2, a phagocytic receptor with still unknown endogenous ligand, leads to activation of microglial cells. Activated microglia in turn can clear cellular apoptotic material, thereby contributing to tissue repair [18, 24, 43]. Therefore, a non-functional TREM2 might be involved in brain damage by causing accumulation of toxic products. Interestingly, loss-of-function mutations of DAP12 or TREM2 both lead to a chronic neurodegenerative disease called Nasu-Hakola or polycystic lipomembranous osteodysplasia with sclerosing leukoencephalopathy (PLOSL), an autosomal recessive inherited disease [18]. While this disease is characterized by early onset presenile dementia followed by delayed bone symptoms in patients carrying TREM2 mutations [44], patients with mutations in DAP12 display an early onset combination of presenile dementia and systemic bone cysts [45, 46]. Moreover, it has been demonstrated that TREM2 is down-regulated by inflammatory signals [47]. All these data indicate that TREM2 might be functionally crucial for the prevention of neurodegenerative processes.

### 4.2. SIRP*β*1

Recently, other new microglial receptors like SIRP*β*1 with a phagocytic ITAM signaling capacity have been identified [25]. Like TREM2, SIRP*β*1 is expressed on microglial cells. In APP-transgenic mice and a mouse model for experimental autoimmune encephalomyelitis, the expression levels of both proteins are increased [25]. Furthermore, TREM2 and SIRP*β*1 are plaque-associated and increase the phagocytic activity of microglia [24, 25, 42, 43]. Upon neurodegenerative signals, TREM2 expression is induced leading to increased phagocytosis and decreased pro-inflammatory responses of microglial cells [42]. However, SIRP*β*1 does not only specifically clear A*β* but also neural debris and microsphere beads [25]. A strong increase of microglial SIRP*β*1 gene transcript has been revealed in the cerebral hemispheres and cerebellum of an animal model of AD, while the gene transcript of DAP12 has only been increased slightly. Thus, up-regulation of SIPR*β*1 does not simply reflect a higher number of microglia. Moreover, it is not directly triggered by the amyloid plaques but by other disease-associated processes including interferons (IFNs) like IFN*β* and IFN*γ*. In cultured microglia, IFNs have been shown to influence the gene transcription of SIRP*β*1 [25]. So far, concrete *in vivo* evidence for a direct pathophysiological relevance of SIRP*β*1 is missing. While SIRP*β*1 could not only be detected on microglial cells associated with plaques, but also in those not directly associated with plaques, it is regarded as a potent regulator of A*β*42 fibril clearance *in vitro* [25].

### 4.3. Complement Receptor 3 (CD11b/CD18)

Complement receptor 3 (CD11b/CD18). Another potential microglial DAP12-associated receptor is the complement receptor 3 (CR3), a major heterodimeric receptor consisting of the integrins CD11b and CD18, which is involved in the complement system. Complement 1q (C1q), the first component of the classical pathway, mediates complement 3 (C3) deposition on apoptotic cells. The phagocytic receptor CR3 plays an important role in the subsequent clearance of C3-opsonized structures [48]. Moreover, sequence similarities to C1q-binding peptides in CD18 suggest direct binding of CR3 (CD11b/CD18) to C1q [49]. As for immunoreceptors, signal transduction by CD18 could follow the ITAM-DAP12 signaling cascade although a direct binding of integrins with ITAM-containing proteins has not been demonstrated so far. But, it has been shown that CD18-mediated Syk activation requires the ITAM-associated molecules DAP12 and FcR*γ* [39]. Furthermore, both DAP12 and CD11b are required for targeted contact of microglia, like for the contact with hippocampal neurons during development that induces cell death [50]. Switching on the complement system plays an important role in initiating inflammatory reactions in the CNS as observed in AD [51] by upregulation of phagocytosis induced via activation and migration of immune cells [52]. Indeed, during formation of amyloid in APP-transgenic mice increased mRNA and protein levels of components of the complement system have been detected. Among those there have been C1q and C3, at which the classical and alternative pathway merge [53]. Moreover, complement activation has been described to occur in amyloid plaques in AD brains [54, 55] and complement products like the membrane attack complex (C5b-9) have been reported to be associated with amyloid plaques [56]. Additionally, C3 seems to be involved in the process of plaque clearance. APP-transgenic mice either deficient in C3 or expressing a C3 complement inhibitor display accelerated A*β* plaque deposition and prominent neurodegeneration [51, 57] as well as a changed activation state of the microglial cells simultaneously [51]. However, while evidences for the direct induction of a phagocytic ITAM signaling by activation of the complement signaling cascade are missing so far, these data suggest an involvement of complement components in microglia for an effective A*β* clearance.

## 5. Microglial Siglecs

Siglecs are members of a subgroup of the Ig superfamily that recognize specific sugar residues on the periphery of cell surface glycans, the sialic acids. Because of their sequence similarity and evolutionary conservation, Siglecs can be separated into two subsets [58]. While CD33-related Siglecs including CD33, Siglec-5 to -11, Siglec-14, and Siglec-16 evolve very rapidly by means of gene duplication or conversion and exon shuffling or loss and show a similarity of ~50–99% in their protein sequences, other members of the Siglec family such as sialoadhesin, CD22, myelin-associated glycoprotein (MAG) and Siglec-15 are more conserved and quite distantly related [27, 40, 59]. Humans display ten CD33-related Siglecs and one Siglec-like protein; mice however express only five CD33-related Siglecs, which seem to have largely lost their CD33-related Siglec genes [27, 40, 59–62]. Siglecs are type 1 transmembrane proteins showing an amino-terminal Ig-like variable (V-set Ig-like) domain that binds sialic acid and variable numbers of Ig-like constant region type 2 (C2-set Ig-like) domains [27, 58, 62, 63]. Mostly, Siglecs function as inhibitory receptors via one or more ITIMs in their cytoplasmic domain [27, 29]. Receptors with ITIMs can counteract signals emanating from ITAM receptors via the recruitment of tyrosine phosphatases such as SHP1 and SHP2 which can lead to the termination of intracellular signals (Figure 1, right side) [27, 59]. Most CD33-related Siglecs, such as the human Siglec-11, are predominantly expressed on mature cells of the immune system such as monocytes and macrophages. Therefore, CD33-related Siglecs are suggested to be important regulators of the innate immunity [27, 59, 62–64]. Siglec-11 has been shown to interact with SHP1 and SHP2 upon tyrosine phosphorylation [29]. Interestingly, SHP1 seems to be involved in antiinflammatory signaling of microglia. Microglia deficient for SHP1 have been demonstrated to produce higher amounts of neurotoxic substances upon LPS stimulation [65]. It has been shown that via interaction of microglial Siglecs with the neuronal glycocalyx microglial neurotoxicity is alleviated. Furthermore, it has been demonstrated that Siglec-11 expressing microglial cells show a reduced phagocytic capacity of apoptotic material in microglia-neuron coculture experiments [66], indicating that ITIM-signaling could be the opponent of the phagocytosis-associated ITAM-Syk signaling pathway [21]. In addition to inhibiting cellular activation, CD33-related Siglecs participate in the induction of apoptosis and the release of pro-inflammatory cytokines [27, 67–69]. However, few Siglecs have been demonstrated to associate with the ITAM-containing adaptor protein DAP12, including the recently discovered human Siglec-16. It contains a positively charged lysine residue in its transmembrane domain but lacks ITIM in its short cytoplasmic tail [40]. Cao et al. [40] have shown that Siglec-16 is expressed on macrophages and on rare microglial-like cell populations in the normal human brain. Phylogenetic analysis of the transmembrane and cytoplasmic tail domain of human and mammalian CD33-related Siglecs revealed that Siglec-16 and the before mentioned Siglec-11 are found in humans, but no direct othologues exist in rodents [40]. This indicates that these two proteins expressed on human myeloid cells could especially be involved in diseases that are uniquely occurring with their whole peculiarities only in humans such as AD. So far, it is not known whether DAP12 associated Siglecs also have a sialic acid binding specificity as observed for the ITIM bearing Siglecs. This should be investigated in the future to find out whether such Siglecs could counter-regulate each other. However, the involvement of different Siglecs in all processes including apoptosis and inflammation indicates a modulatory role of Siglecs in neuroinflammatory and neurodegenerative diseases.

## 6. Conclusion

The biological functions of ITAM-/ITIM-signaling in microglia are not fully understood. Several publications indicate the involvement of ITAM- and ITIM-signaling receptors in CNS innate immune responses and neuroinflammation. It is now becoming evident that those receptors also play a major role in modulating microglial phagocytosis and cytokine expression. Thus, dysfunctional ITAM-/ITIM-signaling receptors lead to chronic neurodegenerative diseases like Nasu-Hakola disease characterized by presenile dementia. These new insights might have important implications for the pathogenesis and treatment of the neuroinflammatory component of neurodegenerative diseases.
